# Development and Evaluation of a Physiologically Based Pharmacokinetic Model of Labetalol in Healthy and Diseased Populations

**DOI:** 10.3390/pharmaceutics14112362

**Published:** 2022-11-02

**Authors:** Hafsa Hafsa, Ammara Zamir, Muhammad Fawad Rasool, Imran Imran, Hamid Saeed, Tanveer Ahmad, Sary Alsanea, Ali A. Alshamrani, Abdullah H. Alruwaili, Faleh Alqahtani

**Affiliations:** 1 Department of Pharmacy Practice, Faculty of Pharmacy, Bahauddin Zakariya University, Multan 60800, Pakistan; 2 Department of Pharmacology, Faculty of Pharmacy, Bahauddin Zakariya University, Multan 60800, Pakistan; 3 Section of Pharmaceutics, University College of Pharmacy, Allama Iqbal Campus, University of the Punjab, Lahore 54000, Pakistan; 4 Institute for Advanced Biosciences (IAB), CNRS UMR5309, INSERM U1209, Grenoble Alpes University, 38700 La Tronche, France; 5 Department of Pharmacology and Toxicology, College of Pharmacy, King Saud University, Riyadh 11451, Saudi Arabia

**Keywords:** labetalol, pharmacokinetics, beta blocker, drug-drug interaction, drug-food interaction, PBPK, hepatic disease, renal failure

## Abstract

Labetalol is a drug that exhibits both alpha and beta-adrenergic receptor-blocking properties. The American Heart Association/American Stroke Association (AHA/ASA) has recommended labetalol as an initial treatment option for the management of severe hypertension. The physiologically based pharmacokinetic (PBPK) model is an in silico approach to determining the pharmacokinetics (PK) of a drug by incorporating blood flow and tissue composition of the organs. This study was conducted to evaluate the primary reasons for the difference in PK after intravenous (IV) and oral administration in healthy and diseased (renal and hepatic) populations. A comprehensive literature search was done using two databases, PubMed and Google Scholar. Various PK parameters were screened for the development of the PBPK model utilizing a population-based PK-Sim simulator. Simulations were performed after creating building blocks firstly in healthy individuals and then in diseased patients after IV and oral administration. The disposition of labetalol after IV and oral administration occurring in patients with the hepatic and renal disease was predicted. The model was evaluated by calculating the R_obs_/_pred_ ratio and average fold error (AFE), which was in the two-fold error range. Moreover, Box-whisker plots were made to compare the overall concentration of the drug in the body at various stages of disease severity. The presented model provides useful quantitative estimates of drug dosing in patients fighting against severe chronic diseases.

## 1. Introduction

Labetalol is a β-adrenergic blocker, that was discovered in 1966, followed by the approval of the Food and Drug Administration (FDA) for medical use in 1977 [1]. It is suitable for the treatment of severe hypertension, pregnancy-induced hypertension, pain induced by angina, heart attack, stroke, and other heart diseases [2]. In the treatment of hypertension, β-adrenergic blockers are thought to be the most suitable first-line alternatives, as stated in the 7th Report of the Joint National Committee on Prevention, Detection, Evaluation, and Treatment of High Blood Pressure (JNC 7) [3]. Labetalol selectively and non-selectively blocks the α-1 adrenergic and β-adrenergic receptors, respectively. It acts by blocking the actions of certain endogenous chemicals, such as adrenaline on the heart and blood vessels, thus lowering heart rate and blood pressure [4]. Labetalol is effective in both oral and parenteral forms. 

Labetalol is categorized as Biopharmaceutics Classification System (BCS) Class 1 [5]. After oral administration, it is readily absorbed and has an absolute bioavailability of approximately 25% [6]. Labetalol is approximately 50–57% bound to plasma proteins [7] and its volume of distribution and elimination half-life is 9–15.7 L/kg and 5.52 h, respectively [8,9]. It is metabolized by a microsomal glycosyltransferase namely uridine 5-disphosphoglucuronosyltransferase (UDP) with no active metabolites [6] being excreted in the urine and feces (through bile). The total body clearance of labetalol is 10–25 mL/min/kg [10]. In the case of oral administration, it has three times the beta-blocking ability than its alpha-blocking ability (i.e., 3:1) while, in the case of intravenous administration the ratio extends to 6.9:1 [11].

Labetalol falls in pregnancy category C [6]. It can cross the placental barrier and only a negligible amount of drug can cross the blood-brain barrier. Its most troublesome side effects are nasal stuffiness, sexual dysfunction, and urinary retention [11]. These side effects can become more severe as the bioavailability of labetalol increases and excretion decreases, especially in cases of liver failure and chronic kidney disease. Therefore, it is highly recommended by the drug authorities to determine the exact dose required in patients fighting against these diseases [12,13]. For this purpose, there is a need for the development of in silico methods, that can assist practitioners in adjusting labetalol dosing in different patients. 

Previously, for pharmacokinetic (PK) analysis, conventional mammillary models were used that were highly dependent on drug quality and had indistinct biological features [14]. However, now for the sake of drug development, a variety of computational models are present. These models represent a mechanistic representation of the drug in the biological system allowing a deductive simulation of drug concentration-time profiles by incorporating information on the drug with little knowledge of the physiological system at the organism level [15]. Teorell firstly described the concept of physiologically-based pharmacokinetic (PBPK) modeling in 1937. PBPK recruits richer informational content compared to the empirical models concerning the anatomy and physiology of the associated system, making it possible to predict drug exposure in unreachable tissues where the drug exerts its toxic or beneficial effects. It also provides beneficial information in predicting the plasma concentration vs. time profiles with the help of preclinical in vitro and in vivo data to support decisions at various drug development stages [16]. In literature, models have been developed previously for drugs in the case of hepatically impaired [17] and chronic kidney disease populations (CKD) [18].

In the case of liver impairment and CKD, many pathophysiological changes occur that can alter the overall PK of labetalol, thus aggravating the related side effects. PBPK modeling provides a platform for the incorporation of these respective changes and in turn determining variations in the PK of the corresponding drug. These include changes in liver volume, hematocrit, protein binding factor, and GFR (in case of liver failure) and hematocrit, plasma protein binding factor, small intestinal transit time, gastrointestinal transit time, and GFR (in case of kidney failure), respectively [19,20]. These changes, when integrated into the model, may assist in optimizing the doses of labetalol in liver impairment and CKD. 

The presented model of labetalol is focused on developing and evaluating the PBPK model for the prediction of PK in the healthy, hepatically impaired, and CKD population by utilizing a methodical approach for model building. To date, no model has been developed on labetalol. The objective of the study is to determine the variations in the labetalol PK after integrating the pathophysiological changes in the CKD and liver failure population, which may aid in providing a deeper understanding and guidance about tailoring drug doses in the respective diseases. 

## 2. Materials and Methods

### 2.1. Literature Review

The systematic analysis was conducted by Cochrane Handbook guidelines to accomplish the study objective and was reported by using the Preferred Reporting Items for Systematic reviews and Meta-analysis (PRISMA) statement [21]. The search was done by using two databases i.e., PubMed and Google Scholar, and the adopted search strategy has been illustrated in supplementary Figure S1. The duplication of all the selected studies was removed by using EndNote X9. The articles related to labetalol in populations such as healthy, renal, liver, hypertension, or pregnancy-induced hypertension and the presence of plasma concentration versus time profile after oral and IV administration, were included in the study. Furthermore, articles were excluded based on title and abstract, the involvement of animals in the study, and if the articles were in open access or not. The details of the sorting of articles are presented in Supplementary Table S1. The units for maximum plasma concentration (C_max_), area under the concentration-time curve (AUC), and clearance (CL), were converted into similar units to make them consistent so that the results can be compared more efficiently. The assessment of the quality of the included studies was carried out by Jadad scoring [22] and the critical appraisal skills Program (CASP) [23]. A detail of Jadad scoring, CASP scoring, and the characteristics of the included studies are given in the Supplementary Tables S2–S4, respectively. Moreover, the details of inclusion and exclusion criteria have been demonstrated in Supplementary Figure S2. From these selected articles only four studies contain plasma-concentration time profiles out of which five profiles of healthy adults and three profiles for disease patients were included in the model development. A detailed description of the selected studies is given below in Table 1.

### 2.2. Modeling Software

The open-system pharmacology suite has been designed to allow efficient modeling and simulation. In this current study, the PBPK modeling of labetalol in healthy adults and diseased (liver and kidney) patients was carried out by using a population-based PK-Sim simulator (version 9.1), which is a component of the computational Systems Biology Software Suite of Bayer Technology Services GmbH (Leverkusen, Germany) and is specifically for whole-body PBPK modeling [28]. 

### 2.3. Building Blocks

The drug and disease-related input data were collected through the literature search conducted previously and the building blocks were created. The non-compartmental analysis (NCA) was performed by scanning data points of plasma-concentration time profiles of all the included studies and importing those data points to the excel sheet. Input parameters that are used in the whole modeling process are compiled in Table 2.

### 2.4. Modeling Strategy

**Figure 1:**
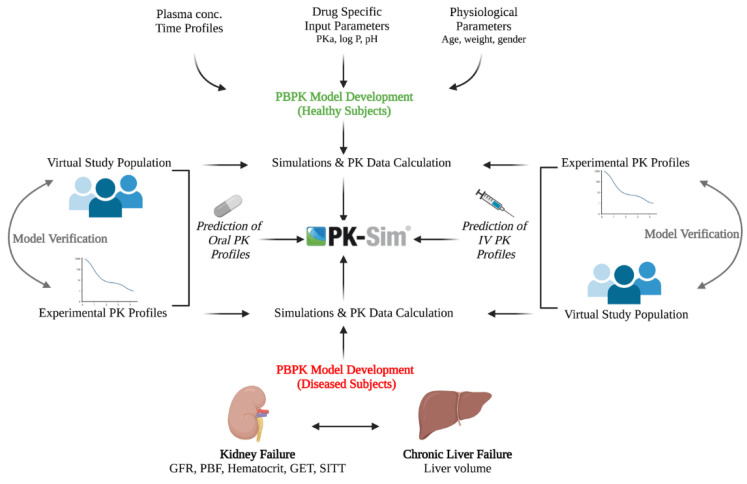
The strategy adopted in the PBPK model of labetalol. GET gastric emptying time, GFR: glomerular filtration rate, IV: intravenous, log P: lipophilicity, pH: the potential of hydrogen, PK: pharmacokinetics, pKa: negative log of the acid dissociation constant, PBF: protein binding factor, SITT; small intestinal Transit time. The figure was created with BioRender.com with agreement number LY24EZHS7O.

The drug disposition behavior was firstly modulated in IV administration after creating the building blocks, and all the parameters related to IV administration were incorporated and simulations were performed. After that, a model for oral administration was modulated as it is a quite complex process compared to IV administration. This complexity is basically due to the distribution of the drug into multiple compartments. For every simulation that was run for a specific study, a total of 100 virtual subjects were created with the corresponding data reported in that specific study. Out of a total of five healthy studies, one-third (1 IV and 1 oral) of studies were used for PBPK model development, whereas the other two-thirds (2 IV and 1 oral) were utilized in model verification. The data include a proportion of females, age (years), weight (kg), height, frequency, and dose administered. The strategy adopted by PBPK modeling has been described below in Figure 1.

### 2.5. Model Parameters

Labetalol (C19H24N2O3) has a pKa value of 9.38, a molecular weight of 328.412 g/mol, and lipophilicity of 2.79 log units, as mentioned previously in Table 2. In PBPK modeling, each organ is recognized by its physiological properties. For orally administered dosage forms, the specific intestinal permeability value of 4.67 × 10^−5^ was incorporated into the model. For the estimation of cellular permeability and tissue plasma partition coefficient, PK-Sim standard model was used. The value of specific organ permeability and specific clearance were optimized using the parameter estimation feature of PK-Sim software to 0.1 L/min and 0.10 L/min respectively. Finally, a total body clearance of 15 ml/min/kg was incorporated into the model (after drawing both oral and IV simulations by using all the values ranging from 10 to 25 mL/min/Kg) based on visual predictive checks and observed/predicted ratios [10].

### 2.6. Model Structure in Diseased Population

#### 2.6.1. Hepatic Disease

Patients with liver cirrhosis often develop different physiological changes that indicate the severity of the disease. Child-Pugh (CP) classification helps to quantify these changes as the disease progresses. These physiological changes include changes in liver volume, hematocrit, plasma protein binding factor, and GFR concerning CP-A, CP-B, and CP-C stages of liver cirrhosis, that has been reported previously [19]. The study selected for the labetalol-cirrhosis model did not mention the CP class of liver cirrhosis therefore separate predictions for every CP class were made and then visually verified by comparing the observed data with the predicted. After that, a labetalol-cirrhosis model for CP-A was developed.

#### 2.6.2. Kidney Disease

The altered physiological function may affect the PK of a drug in patients with kidney disease. To quantitatively predict PK for dose management these physiological changes must be considered when developing a PBPK model in patients with moderate or severe kidney disease and have been reported previously. These reported physiological changes for gastric emptying time, small intestine transit time, GFR, plasma protein binding factor, and hematocrit [20,30]. The selected study has mentioned the severity of kidney disease for creatinine clearance [27]. After calculating and incorporating the respective changes i.e., 24.375 min, 2.94 h, 0.398, 0.843, and 11 mL/min/kg for gastric emptying time, small intestine transit time, hematocrit, plasma protein binding factor, and GFR, respectively, a labetalol-renal failure model for severe kidney failure was developed.

### 2.7. Model Verification

The verification of the current model was done by visual inspection, which was further carried out by comparing the 5th–95th percentile, predicted arithmetic mean, and the minimum and maximum plasma concentration versus time profile with the observed clinical data. The various PK parameters were then determined and the ratio of observed versus predicted (R_obs_/R_pre_) was calculated. Average fold error (AFE) which is the log-transformed ratio of the observed and predictive concentration values, was also calculated to estimate the predictive accuracy of the model for each parameter [31].

Formulas to calculate R_obs_/R_pre_ and AFE are given below:

$$R=\;\frac{\mathrm{Observed}\;\mathrm{value}\;\mathrm{of}\;\mathrm{PK}\;\mathrm{parameter}}{\mathrm{Predicted}\;\mathrm{value}\;\mathrm{of}\;\mathrm{PK}\;\mathrm{parameter}}$$

$$\mathrm{AFE}=10^{\frac{\sum \log\left(\mathrm{fold}\;\mathrm{error}\right)}{N}}$$

### 2.8. Influence of Food and Enzymes on Labetalol PK

From the literature review, it has been noted that enzyme induction and enzyme inhibition may also cause a significant effect on labetalol PK. For this purpose, a study was performed with glutethimide (enzyme inducer) and Cimetidine (enzyme inhibitor) [25], because both of the drugs alter the first-pass metabolism of labetalol and interfere with glucuronide conjugation. Similarly, another study related to food effects on labetalol PK was also reported. This study has documented an increase in the bioavailability of labetalol by decreasing its first-pass metabolism [24].

## 3. Results

The PK parameters after IV and oral administration in healthy IV, oral and diseased populations of all the articles included in this study, and the effect of food and enzymes on labetalol PK, have been stated in Supplementary Tables S5–S9, respectively. 

### 3.1. PBPK Model in Healthy Adults

**Figure 2:**
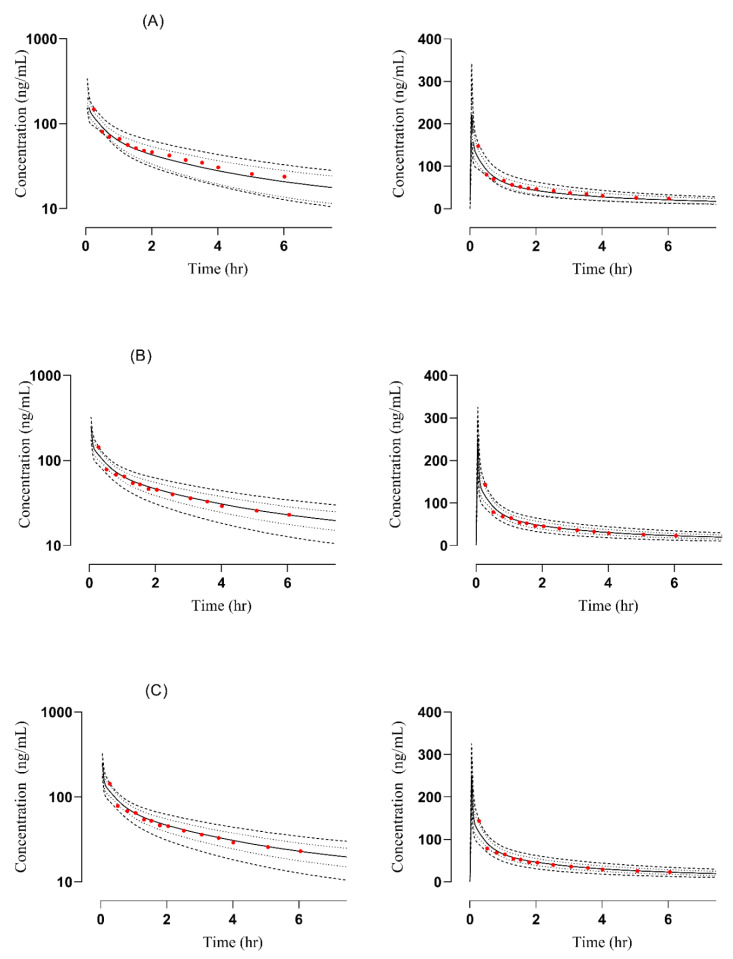
Comparison of observed and predicted plasma concentration versus time profile in healthy subjects after IV administration at a dose of 0.5 mg/kg. (**A**) [24], (**B**) [25]: simulation for labetalol- glutethimide study, (**C**) [25]: simulation for the labetalol-cimetidine study. The solid line [―] indicates the arithmetic mean, dash line [----] maximum and minimum, the dotted line [….] 5th–95th percentile, red dots indicate observed data, and the solid line indicated predicted data.

**Figure 3:**
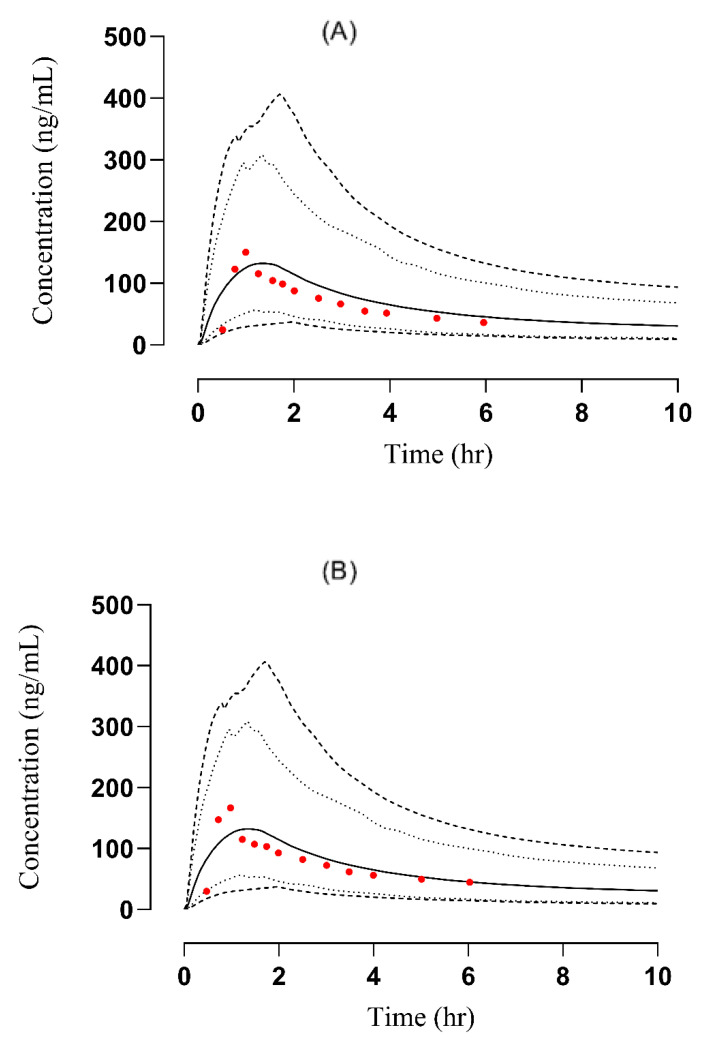
Comparison of observed and predicted plasma concentration versus time profile in healthy subjects after oral administration at a dose of 200 mg. (**A**) [29], (**B**) [25]. The solid line [―] indicates the arithmetic mean, dash line [----] maximum and minimum, the dotted line [….] 5th–95th percentile, red dots indicate observed data, and the solid line indicated predicted data.

After IV (0.5 mg/kg) and oral (200 mg) administration, the plasma concentration versus time profiles of simulated and observed data were compared [24,25]. The observed data sets were visually verified by comparing them with the mean, minimum, maximum, and 5–95 percentile (Figure 2 and Figure 3). The R_obs_/R_pre_ of C_max_, AUC, and CL were calculated (Table 3). The values for C_max_ and CL after IV administration were 1.19–1.60 ng/mL and 0.6–1.2 L/h whereas after oral administration were 1.13–1.26 ng/mL and 0.93–1.18 L/h respectively. To evaluate and determine the accuracy of the PBPK model, the AFE for C_max_, AUC, and CL was also calculated and its values for C_max_ and AUC were 1.44, 1.207, and 1.193, 0.84 for IV and oral administration respectively. A detailed description of AFE calculation has been documented in Supplementary Table S10.

### 3.2. PBPK Model in Diseased Population

#### 3.2.1. Liver Failure 

**Figure 4:**
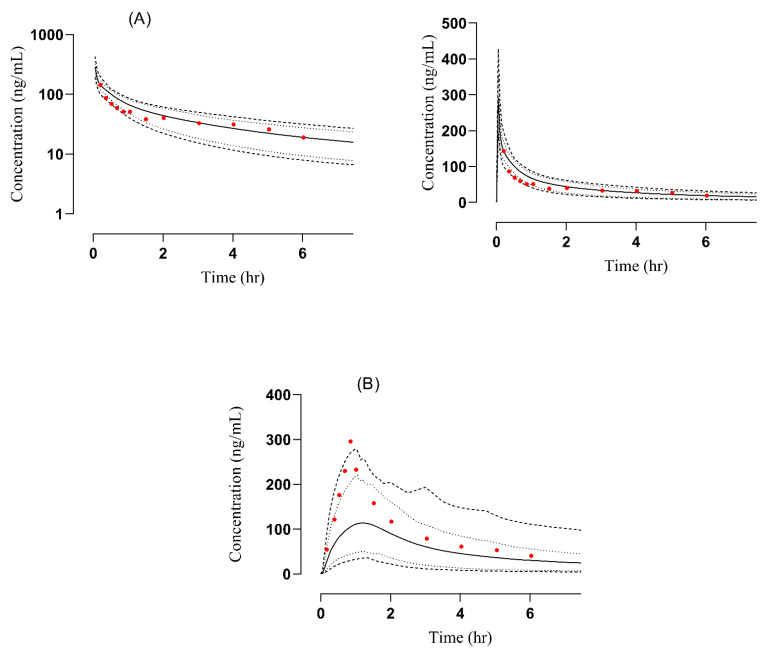
Comparison of observed and predicted plasma versus time profile in hepatic failure subjects after IV (**A**) [26] and oral (**B**) [26] administration at a dose of 0.5 mg/kg and 200 mg, respectively. The solid line [―] indicates the arithmetic mean, dash line [----] maximum and minimum, the dotted line [….] 5th–95th percentile, red dots indicate observed data, and the solid line indicated predicted data.

**Figure 5:**
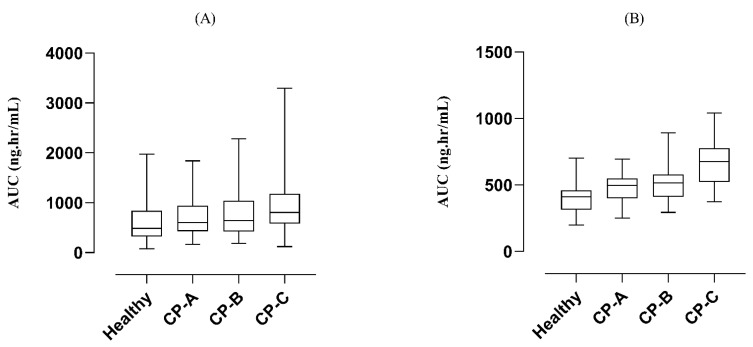
Box-whisker plots after (**A**) oral (200 mg) and (**B**) IV (0.5 mg/kg) dose of labetalol in healthy, CP-A, CP-B, and CP-C populations to compare AUC.

To determine the accuracy of the developed model, the plasma concentration versus time profiles of observed and predicted data were compared (Figure 4). A significant reduction in the values of PK parameters C_max_ from 295.56 ng/mL to 112.14 ng/mL and AUC from 608.73 ng/mL·h to 382.41 ng/mL·h was observed in the case of oral administration compared to the IV administration where a notable elevation i.e., C_max_ from 143.18 ng/ml to 144.19 ng/mL and AUC from 265.30 ng/mL·h to 283.060 ng/mL·h was detected. The resultant R obs/R pre ratio of C_max_ and CL were 0.99 and 1.16 after IV administration whereas 2.635 and 0.6 after oral administration, respectively [26]. All the R_obs_/R_pre_ ratios were in two-fold error range except C_max_ in the case of oral administration (Table 4). This discrepancy in observed and predicted C_max_ is because the study selected for labetalol-liver cirrhosis model development has not mentioned the CP category. Out of the five parameters of the CP classification system only three were mentioned in the study. After carefully assessing the severity of liver cirrhosis and scoring the mentioned parameters, we have considered it to be in CP-A class. Moreover, it can be acceptable but we have to take care of the age, weight, and other parameters while adjusting the doses. The calculated AFE values for C_max_ and CL were 1.009, 1.148, and 2.63, 0.60 after IV and oral administration, respectively. A detail of these calculated values has been added in Supplementary Table S11. Box-whisker plots were made for dose optimization and the AUC was compared for liver cirrhosis after IV administration in which the median along with 95% confidence interval (C.I) for AUC_0–t_ in healthy was 410.4 ng/mL·h (389.0–435.4) which increased to 549.4 ng/mL·h (473.6–511.8) in CP-A, 579.9 ng/mL·h (477.3–543.4) in CP-B and 776.7 ng/mL·h (640.0–722.8) in CP-C whereas after oral administration these values in healthy were 487.8 ng/mL·h (430.4–570.5), which increased to 607.0 ng/mL·h (535.1–707.7) in CP-A, 642.2 ng/mL·h (532.4–795.5) in CP-B and 806.3 ng/mL·h (710.0–924.5) in CP-C, respectively (Figure 5). These changes are associated with a change in AUC as the disease progresses and help in dose adjustment. 

#### 3.2.2. Renal Failure

**Figure 6:**
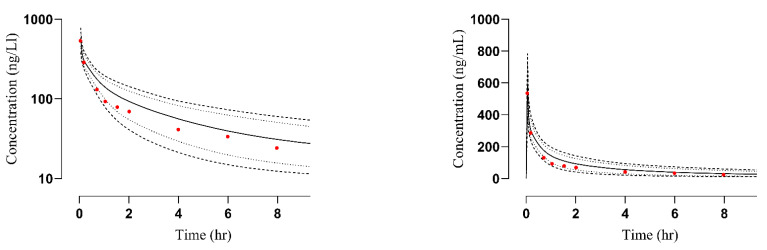
Comparison of observed and predicted plasma concentration versus time profile in renal failure subjects after IV [27] administered at a dose of 1 mg/kg. The solid line [―] indicates the arithmetic mean the dashed line [----] maximum and minimum, the dotted line [….] 5th–95th percentile, red dots indicate observed data and solid line indicated predicted data.

**Figure 7:**
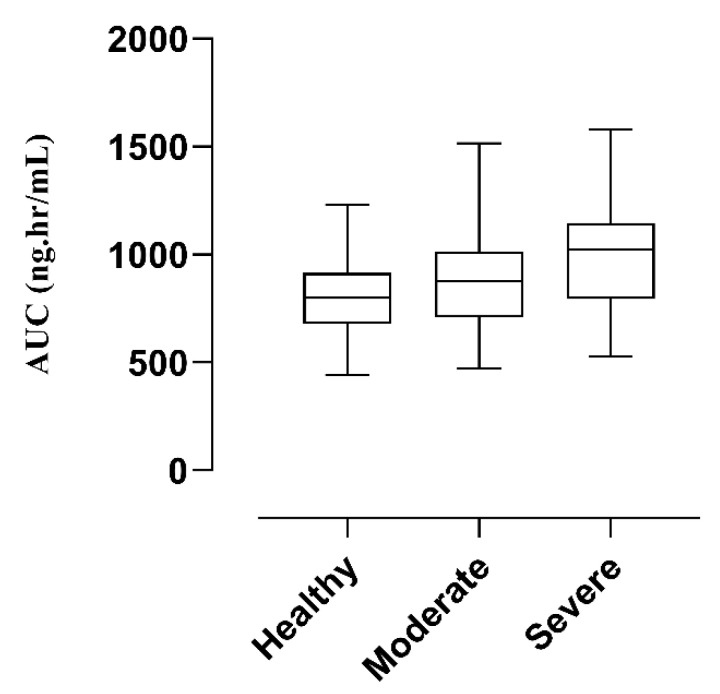
Box-whisker plots after IV (1 mg/kg) dose of labetalol in healthy, moderate kidney failure, and severe kidney failure populations to compare AUC.

The comparison of observed and predicted data points after IV administration is given in Figure 6. A notable increase in the AUC from 590.26 ng/mL·h to 758.806 ng/mL·h and C_max_ from 535.697 ng/mL to 588.037 ng/mL was observed. The calculated R_obs_/R_pre_ values of C_max_ and AUC were 0.91 and 0.77, respectively. These values were in two fold error range and acceptable for the model [27]. A detailed description of the R obs/R pre ratio is given below in Table 4. The AFE values for C_max_ and AUC were 0.912 and 0.77, respectively, and have been documented in Supplementary Table S11. After developing the PBPK model of labetalol in healthy, for dose optimization in patients with kidney disease, Box-whisker plots were made in a sample size comprising 100 subjects (ages: 37–70 years, weight: 75–87 Kg) after IV administration, and the AUC compared in healthy was 799.7 ng/mL·h (740.0–859.7), which increased to 876.9 ng/mL·h (818.3–933.5) in moderate renal failure and 1023 ng/mL·h (952.9–1085) in severe renal failure, respectively (Figure 7). This increase in AUC with an increase in the severity of the disease helps in dose adjustment in such kinds of patients. 

## 4. Discussion

The purpose of the current study was to confine and analyze all the studies published on the PK of labetalol in humans, utilizing a systematic approach and building a PBPK model of labetalol in a healthy population. Once the model was developed, it was extrapolated to the kidney and liver disease population by incorporating physiological changes occurring in these populations as reported in the previous literature. This model has adequately chronicled the disposition of labetalol after oral and IV administration by comparing the predicted plasma concentration versus time profiles of the simulated data, to that of the observed data obtained from the literature. A considerable amount of labetalol passes through extensive first-pass metabolism (glucuronidation) and about 55 to 60% of the drug is eliminated through the urine [5]. A dose-dependent increase was observed in C_max_ and AUC values after oral and IV administration. The values obtained from the current studies dictate a significant difference in IV and oral dose administration related to different PK parameters.

Both the liver and kidney play an important role in the drug PK. The bioavailability of a drug is highly influenced by first-pass metabolism of the liver and the amount of drug absorbed, whereas the elimination of the drug mainly depends on the glomerular filtration rate (GFR). Patients dealing with these incompatibilities may face a reduction in the liver size, hepatic arterial blood flow, albumin, and alpha-1 acid glycoprotein concentration, GFR, cardiac output, gastric emptying time, and small intestinal transit time, which will ultimately make them more prone to the development of irreversible complications [19]. Moreover, as labetalol is indicated in the treatment of hypertension, cardiac blood flow increases according to the Frank-Starling mechanism, but as the arteries become blocked with the severity of diseases, blood flow to the cardiac muscle decreases. Therefore, caution should be taken while adjusting doses of labetalol in hypertensive patients. To determine the degree of liver impairment, CP classification is used for drug dosing, whereas in the case of renal impairment, GFR is highly recommended. It is important to determine these changes which in turn can potentially augment the pharmacological and PK effects of drugs, leading to therapeutic failure or adverse drug effects [19,32].

In the case of chronic liver failure, labetalol kinetics are independent of dose suggesting that the proportionality between AUC and the oral dose is not dose-dependent. This may be due to alteration in the first pass glucuronidation, which can change the bioavailability of labetalol by inducing or inhibiting its metabolic activity [25]. The bioavailability of labetalol increases from 33 to 63% in this condition [26]. Moreover, the drug-metabolizing ability of the liver is also reduced in liver failure because of reduced microsomal enzyme content. The values of T_max_ were similar in both control and patient groups, while the values of C_max_ were observed to be increased in patients as compared to the former group [26]. 

It has been reported that about 55 to 60% of labetalol is eliminated through urine [5]. It means that more than half of the administered drug is removed from the body through the kidneys. Therefore, any physiological change occurring in the kidney function can affect the PK of labetalol leaving the patient more prone to the development of ADRs. This may be due to the decreased excretion of labetalol from the body [27]. The presented model was also extrapolated to the renal failure patients as mentioned previously to compare the observed and predicted PK of labetalol in these patients after IV administration. An increase in the plasma labetalol concentration was observed in these patients, which may be due to a decrease in plasma protein binding associated with an increase in the AUC and a decrease in CL.

It is now clear that patients with liver and renal diseases are more prone to develop complications when taking medicines that are metabolized in the liver and excreted through the kidney. Previously, many studies have been published to determine the changes associated with these diseases [32,33,34,35]. As labetalol is used to treat hypertension it has been reported that labetalol reduces systolic and diastolic blood pressure by about 20%, when administered in a dose of 0.5 to 1 mg/kg [36]. 

The strength of this current study may be that no systematic review and PBPK model has been published on labetalol up till now. Previously, two reviews have been published that were related to the pharmacology and physiological effects of labetalol, respectively. This review has a few limitations. Firstly, only a few studies meet the inclusion criteria and the studies included in the review do not have equal population distribution in both the disease group and the control group, even though some studies have not focused on equal gender proportion. Secondly, in a study related to hepatic disease, all of the five CP parameters were not mentioned, while no plasma concentration versus time profile after oral administration in case of renal failure was assessable to us. Lastly, the results of some important studies were also based on data availability. All of these limitations can decrease the significance of our results.

## 5. Conclusions

All the relevant data about the PK parameters of labetalol have been comprehended in this review. The effect of enzymes, food, chronic liver diseases, and chronic kidney disease on C_max_, T_max_, AUC, and bioavailability have been demonstrated in this review. These results will ultimately help in dose adjustment in patients with chronic liver diseases and chronic kidney disease. Most importantly, the data obtained from this review helped in the development of the PBPK model. The presented model has successfully explained the PK of labetalol after IV and oral administration in healthy and diseased individuals. Moreover, the model is well evaluated by the incorporation of physiological changes occurring in liver and kidney patients in the drug-disease model.

## Figures and Tables

**Table 1:** Characteristics of the studies selected for PBPK modeling.

Sr.	Population	No. of Subjects	Dose	Portion of Females	Age (years)	Weight (kg)	Reference
Oral							
1	Healthy	6	200 mg	2	20–24	N/R	[24]
2	Healthy	5	200 mg	0	21–26	N/R	[25]
IV							
3	Healthy	6	0.5 mg/kg	2	20–24	N/R	[24]
4	Healthy ^a^	5	0.5 mg/kg	0	21–26	N/R	[25]
5	Healthy ^b^	6	0.5 mg/kg	0	21–26	N/R	[25]
Diseased							
6	Chronic liver disease (IV)	10	0.5 mg/kg	2	22–24	50–65	[26]
7	Chronic liver disease (oral)	10	200 mg	2	22–42	50–65	[26]
8	Severe renal failure (IV)	4	1 mg/kg	1	37–70	75–87	[27]

IV: Intravenous, N/R: Not reported, Healthy ^a^: characteristics of subjects selected for labetalol-glutethimide study, Healthy ^b^: characteristics of subjects selected for the labetalol-cimetidine study.

**Table 2:** Input parameters used in PBPK modeling.

Parameters	Value	Reference
Physicochemical parameters		
Molecular weight (g/mol)	328.412	PubChem
pKa	9.38	PubChem
Lipophilicity (log units)	2.79	PubChem
Absorption		
Intestinal permeability (cm/min)	4.67 × 10^−5^	Predicted in PK-Sim
Distribution		
Specific organ permeability (L/min)	0.1	Optimized value ^a^
Fraction unbound (%)	45	[29]
Partition coefficient model	Pk-Sim standard	
Cellular permeability model	Pk-Sim standard	
Elimination		
Total body clearance (mL/min/kg)	15	[10]
Specific clearance (L/h)	6	Optimized value ^a^

^a^ optimized using the parameter estimation feature of the PK-Sim Software.

**Table 3:** Observed and predicted values of various PK parameters and their respective R values in healthy adults.

	C_max_ (ng/mL) ^a^			AUC_0–t_ (ng/mL∙h) ^b^			CL (L/h) ^c^			Reference
IV										
Study	Observed	Predicted	Ratio	Observed	Predicted	Ratio	Observed	Predicted	Ratio	
1	144.244	91.9	1.56	297.002	270.74	1.09	1.2	1	1.2	[24]
2	143.099	120.083	1.19	299.91	288.59	1.039	0.99	1.1	0.9	[25]
3	143.09	89.178	1.60	419.66	279.972	1.498	0.7	1.1	0.63	[25]
Oral										
4	150.228	131.91	1.13	384.602	477.93	0.80	320	270	1.18	[24]
5	166.82	131.91	1.26	432.0	482.57	0.89	244	260	0.93	[25]

C_max_^a^: Maximum plasma concentration, AUC_0–t_ ^b^: Area under the plasma concentration versus time curve, CL ^c^: Clearance.

**Table 4:** Observed and predicted values of various PK parameters and their respective R values in a diseased population.

	C_max_ (ng/mL) ^a^			AUC _0–t_ (ng/mL·h) ^b^			CL (L/h) ^c^			Reference
Study	Observed	Predicted	Ratio	Observed	Predicted	Ratio	Observed	Predicted	Ratio	
Hepatic IV										
1	143.18	144.1995	0.99	265.30	283.060	0.93	1.4	1.2	1.16	[26]
Hepatic Oral										
2	295.567	112.14	2.635	608.733	382.41	1.5	250.6	376	0.6	[26]
Renal IV										
3	535.697	588.037	0.9109	590.26	758.806	0.77	1.4	0.98	1.5	[27]

C_max_^a^: Maximum plasma concentration, AUC_0–t_ ^b^: Area under the plasma concentration versus time curve, CL ^c^: Clearance.

## Data Availability

All the data related to the presented work has been provided in the main text or in the supplementary material.

## Supplementary Materials

The following supporting information can be downloaded at: https://www.mdpi.com/article/10.3390/pharmaceutics14112362/s1, Table S1. Screening of articles based on title, abstract, involvement of animals, and accessibility, Table S2. Quality assessment of included articles using Jadad scoring, Table S3. Quality assessment of included articles using CASP scoring, Table S4. Characteristics of the included studies, Table S5: Pharmacokinetic parameters of the intravenous studies, Table S6: Pharmacokinetic parameters of oral studies, Table S7: Pharmacokinetic parameters of diseased studies, Table S8: Influence of food of the pharmacokinetics of Labetalol, Table S9: Effect of enzyme induction and inhibition on labetalol pharmacokinetics, Table S10: Average fold error (AFE) in a healthy population, Table S11: Average fold error (AFE) in a diseased population, Figure S1. Boolean Operators search strategy, Figure S2. PRISMA flow chat diagram.
