# Supplementary material for: Development and Evaluation of a Physiologically Based Pharmacokinetic Model of Labetalol in Healthy and Diseased Populations
Source: Pharmaceutics. 2022 Nov 2;14(11):2362. doi: 10.3390/pharmaceutics14112362 (PMC9696499; doi:10.3390/pharmaceutics14112362)
Supplement: Supplementary file 1 [file pharmaceutics-14-02362-s001.zip › pharmaceutics-1959501-supplementary.pdf]

Development and Evaluation of a Physiologically Based Pharmacokinetic Model of labetalol in healthy and diseased populations

Hafsa Hafsa [1], Ammara Zamir [1], Muhammad Fawad Rasool [1,*], Imran Imran [2], Hamid Saeed [3], Tanveer Ahmad [4], Sary Alsanea [5], Ali A. Alshamrani [5], Abdullah H. Alruwaili [5] and Faleh Alqahtani [5,*]

[1] Department of Pharmacy Practice, Faculty of Pharmacy, Bahauddin Zakariya University, Multan 60800, Pakistan
[2] Department of Pharmacology, Faculty of Pharmacy, Bahauddin Zakariya University, Multan 60800, Pakistan
[3] Section of Pharmaceutics, University College of Pharmacy, Allama Iqbal Campus, University of the Punjab, Lahore 54000, Pakistan
[4] Institute for Advanced Biosciences (IAB), CNRS UMR5309, INSERM U1209, Grenoble Alpes University, 38700 La Tronche, France
[5] Department of Pharmacology and Toxicology, College of Pharmacy, King Saud University, Riyadh 11451, Saudi Arabia
* Correspondence: fawadrasool@bzu.edu.pk (M.F.R.); afaleh@ksu.edu.sa (F.A.)

Supplementary Table S1. Screening of articles based on title, abstract, involvement of animals, and accessibility

Sr.	Title of article	Exclusion bases
1	Donnelly, R. and G. J. Macphee (1991). "Clinical pharmacokinetics and kinetic-dynamic relationships of dilevalol and labetalol." Clinical pharmacokinetics 21(2): 95-109.	Abstract
2	Ågesen, F. N., et al. (2019). "Pharmacokinetic variability of beta-adrenergic blocking agents used in cardiology." Pharmacol Res Perspect 7(4): e00496.	Title
3	Fongemie, J. and E. Felix-Getzik (2015). "A Review of Nebivolol Pharmacology and Clinical Evidence." Drugs 75(12): 1349-1371.	Title
4	Kaye, A. B., et al. (2019). "Review of Cardiovascular Drugs in Pregnancy." J Womens Health (Larchmt) 28(5): 686-697.	Title
5	Yeleswaram, K., et al. (1992). "Pharmacokinetics and pharmacodynamics of labetalol in the pregnant sheep." J Pharmacol Exp Ther 262(2): 683-691.	Animal
6	Fischer, J. H., et al. (2014). "Influence of gestational age and body weight on the pharmacokinetics of labetalol in pregnancy." Clin Pharmacokinet 53(4): 373-383.	Abstract
7	Mulrenin, I. R., et al. (2021). "The Impact of Pregnancy on Antihypertensive Drug Metabolism and Pharmacokinetics: Current Status and Future Directions." Expert Opin Drug Metab Toxicol 17(11): 1261-1279.	Title
8	Clark, S. M., et al. (2015). "A review of oral labetalol and nifedipine in mild to moderate hypertension in pregnancy." Semin Perinatol 39(7): 548-555.	Abstract
9	Rawat, P. S., et al. (2020). "Development and validation of a bio-analytical method for simultaneous quantification of nebivolol and labetalol in aqueous humor and plasma using LC-MS/MS and its application to ocular pharmacokinetic studies." J Chromatogr B Analyt Technol Biomed Life Sci 1136: 121908.	Title
10	McNeil, J. J. and W. J. Louis (1984). "Clinical pharmacokinetics of labetalol." Clin Pharmacokinet 9(2): 157-167.	Abstract
11	Sanders, G. L., et al. (1980). "Pharmacokinetics, beta-adrenoceptor blockade and anti-hypertensive action of labetalol during chronic oral treatment." Br J Clin Pharmacol 10(2): 121-126.	Abstract

Sr.	Title of article	Exclusion bases
12	Rocci, M. L., Jr., et al. (1990). "Pharmacokinetics and pharmacodynamics of labetalol in elderly and young hypertensive patients following single and multiple doses." Pharmacotherapy 10(2): 92-99.	Abstract
13	Chera-Aree, P., et al. (2020). "Clinical Experiences of Intravenous Hydralazine and Labetalol for Acute Treatment of Severe Hypertension in Pregnant Thai Women." J Clin Pharmacol 60(12): 1662-1670.	Title
14	Awni, W. M., et al. (1988). "Interindividual and intraindividual variability in labetalol pharmacokinetics." J Clin Pharmacol 28(4): 344-349.	Abstract
15	Sonne, J., et al. (1990). "Single dose pharmacokinetics and pharmacodynamics of oral oxazepam during concomitant administration of propranolol and labetalol." Br J Clin Pharmacol 29(1): 33-37.	No access
16	Alshami, A., et al. (2018). "Management of hypertensive crises in the elderly." J Geriatr Cardiol 15(7): 504-512.	Abstract
17	McNeil, J. J., et al. (1979). "Pharmacokinetics and pharmacodynamic studies of labetalol in hypertensive subjects." Br J Clin Pharmacol 8(Suppl 2): 157s-161s.	Abstract
18	Incecayir, T., et al. (2013). "Comparison of the permeability of metoprolol and labetalol in rat, mouse, and Caco-2 cells: use as a reference standard for BCS classification." Mol Pharm 10(3): 958-966.	Animal
19	Nylund, L., et al. (1984). "Labetalol for the treatment of hypertension in pregnancy. Pharmacokinetics and effects on the uteroplacental blood flow." Acta Obstet Gynecol Scand Suppl 118: 71-73.	Title
20	Hermann, D. J., et al. (1992). "Comparison of verapamil, diltiazem, and labetalol on the bioavailability and metabolism of imipramine." J Clin Pharmacol 32(2): 176-183.	Abstract
21	Riva, E., et al. (1991). "The alpha- and beta-adrenoceptor blocking activities of labetalol and its RR-SR (50:50) stereoisomers." Br J Pharmacol 104(4): 823-828.	Abstract
22	Carvalho, T. M., et al. (2009). "Stereoselective analysis of labetalol in human plasma by LC-MS/MS: application to pharmacokinetics." Chirality 21(8): 738-744.	Title
23	Kanto, J., et al. (1981). "Pharmacokinetics of labetalol in healthy volunteers." Int J Clin Pharmacol Ther Toxicol 19(1): 41-44.	No access
24	Dong, S., et al. (2016). "Graphene Facilitated Removal of Labetalol in Laccase-ABTS System: Reaction Efficiency, Pathways and Mechanism." Sci Rep 6: 21396.	Title
25	Abernethy, D. R., et al. (1987). "Comparison in young and elderly patients of pharmacodynamics and disposition of labetalol in systemic hypertension." Am J Cardiol 60(8): 697-702.	Title
26	Yeleswaram, K., et al. (1993). "Transplacental and nonplacental clearances, metabolism and pharmacodynamics of labetalol in the fetal lamb after direct intravenous administration." J Pharmacol Exp Ther 267(1): 425-431.	Animal
27	Yeleswaram, K., et al. (1993). "Disposition, metabolism, and pharmacodynamics of labetalol in adult sheep." Drug Metab Dispos 21(2): 284-292.	Animal
28	Kanto, J. H. (1985). "Current status of labetalol, the first alpha- and beta-blocking agent." Int J Clin Pharmacol Ther Toxicol 23(11): 617-628.	Abstract
29	Bates, J., et al. (1987). "Combined use of an automated sample processor and a polymer-based high-performance liquid chromatographic column to determine the pharmacokinetics of labetalol in man." J Chromatogr 395: 455-461.	Title
30	Abushammala, I., et al. (2006). "Labetalol absorption kinetics: rat small intestine and colon studies." J Pharm Sci 95(8): 1733-1741.	Animal
31	Daneshmend, T. K. and C. J. Roberts (1981). "Cimetidine and bioavailability of labetalol." Lancet 1(8219): 565.	Title
32	Chauvin, M., et al. (1987). "Continuous i.v. infusion of labetalol for postoperative hypertension. Haemodynamic effects and plasma kinetics." Br J Anaesth 59(10): 1250-1256.	Title

Sr.	Title of article	Exclusion bases
33	Li, P., et al. (2012). "Hepatocellular necrosis, fibrosis and microsomal activity determine the hepatic pharmacokinetics of basic drugs in right-heart-failure-induced liver damage." Pharm Res 29(6): 1658-1669.	Title
34	Louis, W. J., et al. (1984). "Pharmacology of combined alpha-beta-blockade. I." Drugs 28 Suppl 2: 16-34.	Title
35	Khafagi, F. A., et al. (1989). "Labetalol reduces iodine-131 MIBG uptake by pheochromocytoma and normal tissues." J Nucl Med 30(4): 481-489.	Title
36	Kouoh, F., et al. (2004). "In vitro and ex vivo antioxidant activities of labetalol on rabbit neutrophil respiratory burst." Adv Ther 21(3): 178-185.	Animal
37	Chrisp, P. and K. L. Goa (1990). "Dilevalol. A review of its pharmacodynamic and pharmacokinetic properties, and therapeutic potential in hypertension." Drugs 39(2): 234-263.	Title
38	Scalzo, F. M., et al. (1993). "Effects of labetalol on cocaine pharmacokinetics in neonatal piglets." Dev Pharmacol Ther 20(1-2): 54-63.	Animal
39	McTavish, D., et al. (1993). "Carvedilol. A review of its pharmacodynamic and pharmacokinetic properties, and therapeutic efficacy." Drugs 45(2): 232-258.	Title
40	Johnson, J. A., et al. (2000). "Gender differences in labetalol kinetics: importance of determining stereoisomer kinetics for racemic drugs." Pharmacotherapy 20(6): 622-628.	Abstract
41	Singh, B. N., et al. (1985). "Acebutolol. A review of its pharmacological properties and therapeutic efficacy in hypertension, angina pectoris and arrhythmia." Drugs 29(6): 531-569.	Title
42	Drop, L., et al. (1998). "Comparative doses and cost: esmolol versus labetalol during electroconvulsive therapy." Anesth Analg 86(4): 916-917.	Title
43	Alton, K. B., et al. (1994). "Urinary metabolites of (R),(R)-labetalol." Drug Metab Dispos 22(6): 866-872.	Abstract
44	Hosey, C. M. and L. Z. Benet (2015). "Predicting the extent of metabolism using in vitro permeability rate measurements and in silico permeability rate predictions." Mol Pharm 12(5): 1456-1466.	Title
45	Radwanski, E., et al. (1988). "Secretion of dilevalol in breast milk." J Clin Pharmacol 28(5): 448-453.	Title
46	Klotz, U. and I. Reimann (1983). "[Effect of histamine H2-receptor antagonists on the hepatic elimination of drugs]." Klin Wochenschr 61(13): 625-632.	Title
47	Shen, J. J., et al. (1991). "Response to beta-blockers in maternal and fetal rat hearts in vitro." Life Sci 48(18): 1737-1743.	Animal
48	Schneider, H. and M. Proegler (1988). "Placental transfer of beta-adrenergic antagonists studied in an in vitro perfusion system of human placental tissue." Am J Obstet Gynecol 159(1): 42-47.	Title
49	Haraldsson, A. and W. Geven (1989). "Half-life of maternal labetalol in a premature infant." Pharm Weekbl Sci 11(6): 229-231.	No access
50	Baba, T., et al. (1988). "Effects of dilevalol, an R, R-isomer of labetalol, on blood pressure and renal function in patients with mild-to-moderate essential hypertension." Eur J Clin Pharmacol 35(1): 9-15.	Title
51	Keski-Rahkonen, P., et al. (2007). "Determination of tamsulosin in human aqueous humor and serum by liquid chromatography-electrospray ionization tandem mass spectrometry." J Pharm Biomed Anal 43(2): 606-612.	Title
52	Gasparović, V., et al. (1988). "[Pharmacokinetics of labetalol in patients on hemodialysis]." Acta Med Iugosl 42(2): 141-145.	Abstract
53	Wang, W., et al. (1991). "Lipophilicity influence on conjunctival drug penetration in the pigmented rabbit: a comparison with corneal penetration." Curr Eye Res 10(6): 571-579.	Animal
54	Thorsteinsson, A., et al. (2008). "Severe labetalol overdose in an 8-month-old infant." Paediatr Anaesth 18(5): 435-438.	Title
55	Adamska-Dyniewska, H., et al. (1986). "The effect of six beta-adrenolytics and labetalol on hepatic biotransformation studied by antipyrine test, in man." Int J Clin Pharmacol Ther Toxicol 24(6): 303-307.	Title

Sr.	Title of article	Exclusion bases
56	Kandasamy, K., et al. (2011). "Bioanalytical method development, validation and quantification of flupirtine maleate in rat plasma by liquid chromatography-tandem mass spectrometry." Arzneimittelforschung 61(12): 693-699.	Animal
57	Nielsen, H. M. and M. R. Rassing (2000). "TR146 cells grown on filters as a model of human buccal epithelium: IV. Permeability of water, mannitol, testosterone and beta-adrenoceptor antagonists. Comparison to human, monkey and porcine buccal mucosa." Int J Pharm 194(2): 155-167.	Title
58	Partridge, B. L., et al. (1988). "Life-threatening effects of intravascular absorption of PGF2 alpha during therapeutic termination of pregnancy." Anesth Analg 67(11): 1111-1113.	Title
59	Grellet, J., et al. (1994). "Sensitive high-performance liquid chromatographic method for the determination of labetalol diastereoisomers in plasma samples without derivatization." J Chromatogr 652(1): 59-66.	Title
60	Cubeddu, L. X., et al. (1987). "Mechanism of the vasodilatory effect of carvedilol in normal volunteers: a comparison with labetalol." J Cardiovasc Pharmacol 10 Suppl 11: S81-84.	No access
61	Villarroya, M., et al. (1997). "Distinct effects of omega-toxins and various groups of Ca(2+)-entry inhibitors on nicotinic acetylcholine receptor and Ca2+ channels of chromaffin cells." Eur J Pharmacol 320(2-3): 249-257.	Title
62	Aqil, M., et al. (2005). "Transdermal drug delivery of labetolol hydrochloride: system development, in vitro; ex vivo and in vivo characterization." Curr Drug Deliv 2(2): 125-131.	Title
63	Meredith, P. A., et al. (1981). "The determination of labetalol in plasma by high-performance liquid chromatography using fluorescence detection." J Pharmacol Methods 6(4): 309-314.	Title
64	Stupack, D. G., et al. (1999). "Heterogeneity among beta-adrenoreceptor blockers in the modulation of energy-dependent uptake of the organic cation amantadine by rat renal tubules." Can J Physiol Pharmacol 77(6): 407-413.	Title
65	Kusala, S., et al. (1987). "[Relative biological availability of labetalol after the administration of the Trandate (Allen & Hanburys) and Coreton (VULM) preparations]." Cesk Fysiol 36(1): 78-85.	Title
66	Catapano, M. S. and J. A. Marx (1986). "Management of urgent hypertension: a comparison of oral treatment regimens in the emergency department." J Emerg Med 4(5): 361-368.	Title
67	Doroudian, A., et al. (1993). "Sensitive high-performance liquid chromatographic method for direct separation of labetalol stereoisomers in biological fluids using an alpha 1-acid glycoprotein stationary phase." J Chromatogr 619(1): 79-86.	Title
68	Mayer, S., et al. (2000). "Influence of drugs on myocardial iodine-123 metaiodobenzylguanidine uptake in rabbit myocardium." Eur J Nucl Med 27(3): 340-345.	Animal
69	Weissman, C., et al. (1995). "Do synthetic adrenergic agents interfere with the measurement of endogenous plasma catecholamine concentrations?" J Crit Care 10(2): 72-77.	Title
70	Hu, O. Y., et al. (1991). "Determination of vecuronium in blood by HPLC with UV and electrochemical detection: a pilot study in man." Proc Natl Sci Counc Repub China B 15(3): 186-190.	Title
71	Kinami, J., et al. (1993). "Nipradilol displays a unique pharmacological profile of affinities for the different alpha 1-adrenoceptor subtypes." Jpn J Pharmacol 61(2): 81-86.	Title
72	Huguet, F., et al. (1996). "Interaction of metaiodobenzylguanidine with cardioactive drugs: an in vitro study." Eur J Nucl Med 23(5): 546-549.	Title
73	Donaghy, S. (2005). "Choice of antihypertensives after acute ischemic stroke." Cmaj 173(4): 340; author reply 340.	Title
74	Sala, X., et al. (1993). "[Cardiac arrest in newborn of mother treated with labetalol]." Rev Esp Anestesiol Reanim 40(3): 146-147.	Title
75	Fujimura, A., et al. (1989). "Clinical pharmacology of dilevalol (III). A pharmacokinetic study of dilevalol in elderly subjects with essential hypertension." J Clin Pharmacol 29(11): 1008-1012.	Title

Sr.	Title of article	Exclusion bases
76	Kelly, J. G., et al. (1990). "The pharmacokinetics of dilevalol in renal impairment." J Hum Hypertens 4 Suppl 2: 59-62.	Title
77	Oosterhuis, B., et al. (1981). "HPLC-analysis and preliminary pharmacokinetic parameter estimations of chloroquine." Pharm Weekbl Sci 3(6): 263-267.	Title
78	Mann, H. J. and R. J. Klecker (1986). "Cost analysis of substituting labetalol for nitroprusside." Am J Hosp Pharm 43(6): 1501-1502.	Title
79	Adamska-Dyniewska, H. and S. Dziekański (1984). "[Effect of labetalol on antipyrine metabolism in the liver]." Pol Arch Med Wewn 71(3): 149-155.	Title
80	Sun, C.-J., et al. (2018). "Associations of polymorphisms of CYP2D6 and CYP2C9 with early onset severe pre-eclampsia and response to labetalol therapy." Archives of Gynecology and Obstetrics 298(1): 125-132.	Title
81	Jeong, H., et al. (2008). "Regulation of UDP-glucuronosyltransferase (UGT) 1A1 by progesterone and its impact on labetalol elimination." Xenobiotica 38(1): 62-75.	Abstract
82	Olsen, K., et al. (1995). "Effect of labetalol on cerebral blood flow, oxygen metabolism and autoregulation in healthy humans." British Journal of Anaesthesia 75(1): 51-54.	Abstract
83	Saotome, T., et al. (1993). "Labetalol in Hypertension During the Third Trimester of Pregnancy: Its Antihypertensive Effect and Pharmacokinetic-Dynamic Analysis." The Journal of Clinical Pharmacology 33(10): 979-988.	Title
84	Alton, K., et al. (1984). "High-performance liquid chromatographic assay for labetalol in human plasma using a PRP-1 column and fluorometric detection." Journal of Chromatography B: Biomedical Sciences and Applications 311: 319-328.	Title
85	Doroudian, A. (2000). Pharmacokinetics and conjugative metabolism of labetalol stereoisomers in pregnant sheep: A chiral drug case study in pregnancy, National Library of Canada= Bibliothèque nationale du Canada, Ottawa.	Animal
86	Wallin, J. D. and W. M. O'Neill (1983). "Labetalol: Current research and therapeutic status." Archives of internal medicine 143(3): 485-490.	Abstract
87	Scott, A. K. (1993). "Stereoisomers and drug toxicity." Drug safety 8(2): 149-159.	Title
88	van Zwieten, P. A. (1993). "An overview of the pharmacodynamic properties and therapeutic potential of combined α-and β-adrenoceptor antagonists." Drugs 45(4): 509-517.	Abstract
89	Pagliara, A., et al. (1998). "Molecular properties and pharmacokinetic behavior of cetirizine, a zwitterionic H1-receptor antagonist." Journal of medicinal chemistry 41(6): 853-863.	Title
90	Wang, Y. H., et al. (2012). "UGT2B17 genetic polymorphisms dramatically affect the pharmacokinetics of MK-7246 in healthy subjects in a first-in-human study." Clinical Pharmacology & Therapeutics 92(1): 96-102.	Title
91	Wood, A. J. and H. H. Zhou (1991). "Ethnic differences in drug disposition and responsiveness." Clinical pharmacokinetics 20(5): 350-373.	Title
92	van der Galiën, R., et al. (2019). "Pharmacokinetics of HIV-integrase inhibitors during pregnancy: mechanisms, clinical implications and knowledge gaps." Clinical pharmacokinetics 58(3): 309-323.	Title
93	Marasanapalle, V. P., et al. (2011). "Correlation between the systemic clearance of drugs and their food effects in humans." Drug Development and Industrial Pharmacy 37(11): 1311-1317.	Title
94	Fokina, V. M., et al. (2016). "Pharmacokinetics of bupropion and its pharmacologically active metabolites in pregnancy." Drug Metabolism and Disposition 44(11): 1832-1838.	Title
95	Yang, L., et al. (2021). "Metabolic Activation and Cytotoxicity of Labetalol Hydrochloride Mediated by Sulfotransferases." Chemical Research in Toxicology 34(6): 1612-1618.	Title

Supplementary Table S2. Quality assessment of included articles using Jadad scoring

Sr	Title of Article	Jadad Scoring
1	Single–dose pharmacokinetics of labetalol in healthy young men	2+0+0=2
2	Clinical pharmacology of dilevalol (I). Comparison of the pharmacokinetic and pharmacodynamic properties of dilevalol and labetalol after a single oral administration in healthy subjects	2+2+0=4
3	Comparison of the clinical pharmacokinetics and concentration-effect relationships for medroxalol and labetalol.	2+2+0=4
4	Labetalol pharmacokinetics and pharmacodynamics: evidence of stereoselective disposition.	2+2+0=4
5	Pharmacokinetic interaction studies of atosiban with labetalol or betamethasone in healthy female volunteers	2+0+0=2
6	The influence of food on the oral and intravenous pharmacokinetics of a high clearance drug: a study with labetalol	2+2+1=5
7	Decreased first-pass metabolism of labetalol in chronic liver disease.	2+2+0=4
8	Elimination kinetics of labetalol in severe renal failure	2+0+0=2
9	The effects of enzyme induction and enzyme inhibition on labetalol pharmacokinetics	2+2+0=4
10	Rising multiple-dose pharmacokinetics of labetalol in hypertensive patients.	2+2+1=5

Supplementary Table S3. Quality assessment of included articles using CASP scoring

**Questions**	Did the study address a clearly issue?	Did the authors use an appropriate method to answer their questions?	Was the data collected from a clear and suitable source?	Were the controls selected in an acceptable way?	Did the authors dedicate a means to avoid bias?	Have the authors taken account of the potential confounding factors in the design and/or their analysis?	Are the results clear to the reader?	Are the results precise?	Is the model validated?	Is the model applicable to the general population?	Did the results fit with other available evidence?	Score out of 11
Nyberg et al 1982	Y	Y	Y	Y	Y	CT	Y	N	N	N	CT	6
Fujimura et al (1989)	Y	Y	Y	Y	N	CT	Y	N	N	N	N	5
Elliott et al (1984)	Y	Y	CT	CT	N	CT	Y	N	N	N	CT	3
Lalonde et al (1990)	Y	Y	Y	N	N	CT	N	CT	CT	N	N	3
Rasmussen et al (2005)	Y	Y	Y	Y	N	CT	Y	Y	N	Y	N	7
Daneshmend et al (1982)	Y	Y	Y	N	N	CT	Y	N	N	N	N	4
Homeida et al (1978)	Y	Y	CT	N	N	CT	Y	N	N	CT	N	3
Wood et al (1982)	Y	Y	Y	N	N	CT	Y	Y	N	CT	N	5
Daneshmend et al (1984)	Y	Y	Y	Y	N	CT	N	Y	Y	Y	Y	8
Chung et al (1986)	Y	N	N	N	N	CT	Y	N	CT	N	N	2
Y=Yes, N=No, CT=Can't tell												

Supplementary Table S4. Characteristics of the included studies

Sr. No.	Author	Population	N[a]	Age (years)	Weight (kg)	Dosage form		Dose		Frequency
						Tab[b]	Inj[c]	Oral (mg)	IV[d] (mg/kg)	
1-	Gunnar nyberg et.al [23]	Healthy	5	28–46	73–96	Tab	Inj	200	200*	OD[e]
								400		
2-	Akio fujimura et.al [24]	Healthy	6	22–38	56–74	Tab		100		OD
3-	H. L. Ellioyt et.al [25]	Healthy	9	21–38		Tab	Inj	400	1	OD
4-	Richard l et.al [26]	Healthy	9	22–32	57–100	Tab	Inj	200	1.2	BD[f]
5-	Birgitte buur rasmussen, et.al [27]	Healthy	14	18–45	51–75	Tab		100		BD
6-	T.k. daneshmend & c.j.c. Roberts et.al [28]	Healthy	6	20–24		Tab	Inj	200	0.5	OD
7-	M homeida et.al [29]	Healthy	7	22–42	71.4 ± 3.3	Tab	Inj	100	0.5	OD
8-	A.j. wood* et.al [30]	Healthy	3	25–37			Inj		1	OD
9-	T. K. Daneshmend et.al [31]	Healthy	5	21–26		Tab	Inj	200	0.5	OD
			6			Tab		400		QD[g]
10-	M homeida et.al [29]	Diseased	10		74.6 ± 4.8	Tab	Inj	100	0.5	OD
11-	A.j. wood* et.al [30]	Diseased	4	37–70			Inj		1	OD
12-	Menger chung et.al [32]	Diseased	12	27–65		Tab		100		BD
								200		
								300		
								400		
								600		

Tab[b]: Tablet, IV[d]: Intravenous, Inj[c]: Injection, OD[e]: Once daily, BD[f]: Twice daily,

QD[g] : Four times daily N[a:] number of population. * Dose is in mg.

Supplementary Table S5: Pharmacokinetic parameters of the intravenous studies

Sr. No.	Author	Dose (mg/kg)	Vd[a] (L)	C_{max}[b] (ng/ml)	T_{max}[c] (min)	AUC[d] (ng. min/ml)	t ½[e] (hr)	F[f] %	CL[g] (L/hr)
1-	T.k. daneshmend et.al [28]	0.5	567 ± 63	182 ± 57	85 ± 17	23986 ± 4307	3.5 ± 0.4	0.26 ± 0.03	115.3
2-	M homeida et.al [29]	0.5	805 ± 91			14986 ± 3258	3.1 ± 0.4	33 ± 3	
3-	T. K. Daneshmend et.al [31]	0.5	542 ± 72			24438 ± 5237	3.4 ± 0.5	30 ± 3	116.4 ± 30.5
		0.5	520 ± 51	338 ± 30		34924 ± 2288	5.7 ± 0.75	25 ± 2	63.72 ± 6.9
4-	H. L. Ellioyt et.al [25]	1		327 ± 150	66 ± 18	52560 ± 26820	5.2 ± 1.3	20 ± 5	93.6 ± 38.8
5-	Richard l. Lalonde et.al[26]	1.2		274 ± 99		50100 ± 29760	8.23 ± 1.38	44 ± 14	109.11 ± 24.3
6-	A.j. wood et.al [30]	1	167.48–237				3.1–6.9		16.43–49.6

Vd[a]: Volume of distribution, C_{max}[b]: Maximum plasma concentration, T_{max}[c] : Time to reach maximum plasma concentration, AUC[d]: Area under the plasma concentration-time curve, $t_{1/2}$[e]: Half-life, F[f]: bioavailability, CL[g]: Clearance

Supplementary Table S6: Pharmacokinetic parameters of oral studies

Sr. No.	Author	Dose (mg)	Vd[a](L)	C_{max}[b] (ng/ml)	T_{max}[c] (min)	AUC[d] (ng min /ml)	$t_{1/2}$[e] (hr)	F[f] %	CL[g] (L/hr)
1-	Gunnar nyberg et.al [23]	200	823		30–120		1.6–8.5 hr		52.2 (mean)*
2-	Akio fujimura et.al [24]	100		40.5 ± 4.7	48 ± 6	9408 ± 1398	0.74 ± 0.23		
3-	H. L. Ellioyt et.al [25]	400		327 ± 150	66 ± 18	52560 ± 26820	5.5 ± 5.0	20 ± 5	93.6 ± 38.88
4-	Richard l. Lalonde et.al [26]	200		274 ± 99		50100 ± 29760	8.27±1.38	44 ± 14	276.47 ±109.7
5-	Birgitte buur rasmussen et.al [27]	100				34260			
6-	T.k. daneshmend et.al [28]	200	567 ± 63	182 ± 57	85 ± 17	36032 ± 10480	3.06 ± 0.4	26 ± 3	115.38 ± 25.02
7-	M homeida et.al [29]	200	805 ± 91			16002 ± 2665	2.56 ± 0.55	33 ± 3	
8-	T. K. Daneshmend et.al [31]	200	542 ± 72			40596 ± 11534	3.21 ± 0.31	30±3	116.4 ± 30.54
		400	520 ± 59			51029 ± 7950	4.3 ± 0.3	25 ± 2	63.72 ± 6.96

Vd[a]: Volume of distribution, C_{max}[b]: Maximum plasma concentration, T_{max}[c] : Time to reach maximum plasma concentration,

AUC[d]: Area under the plasma concentration-time curve,

$t_{1/2}$[e]: Half-life, F[f]: bioavailability, CL[g]: Clearance, * value of clearance is in mean.

Supplementary Table S7: Pharmacokinetic parameters of diseased studies

Sr. No.	Author	Dose	Vd^a (L)	C_{max}^b (ng/ml)	T_{max}^c (min)	AUC^d(ng.min/ml)	$t_{1/2}^e$ (hr)	F^f %	CL^g (L/hr)
**Oral**									
1-	M homeida et.al [29]	200 mg	526 ± 31			47640 ± 9040	2.6 ± 0.3	63 ± 7	
2-	Menger chung et.al [32]	100 mg		81 ± 47	78 ± 30	27060 ± 16740	8		
		200 mg		218 ± 96	72 ± 24	66120 ± 33720			
		300 mg		214 ± 97	90 ± 54	81900 ± 42600			
		400 mg		466 ± 214	66 ± 18	130140 ± 66360			
		600 mg		642 ± 379	90 ± 30	226260 ± 133500			
**Intravenous**									
3-	A.j. wood et.al [30]	1 mg/kg	175–421				4.1–7.4		15.9–63.96
4-	M homeida et.al [29]	0.5 mg/kg	526 ± 31			18646 ± 2788	2.8 ± 0.4		

Vd^a: Volume of distribution, C_{max}^b: Maximum plasma concentration, T_{max}^c: Time to reach maximum plasma concentration, AUC^d: Area under the plasma concentration-time curve, $t_{1/2}^e$: Half-life, F^f: bioavailability, CL^g: Clearance

Supplementary Table S8: Influence of food of the pharmacokinetics of Labetalol

	Vd[a] (L)	C_{max}[b] (ng/ml)	T_{max}[c](min)	AUC[d] (ng.min/ml)		$t_{1/2}$[e] (hr)		F[f] %	CL[g] (L/hr)
				Oral	IV[h]	Oral	IV		
Fasting State:	567 ± 63	182 ± 57	85 ± 17	36032 ± 10480	23986 ± 4307	3.0 ± 0.4	3.5 ± 0.4	0.26 ± 0.03	115.38 ± 25.02
Fed State:	685 ± 76	180 ± 33	125 ± 9	40085 ± 11448	19887 ± 1412	3.0 ± 0.5	3.6 ± 0.4	0.36 ± 0.05	140.64 ± 33.96

Vd[a]: Volume of distribution, C_{max}[b]: Maximum plasma concentration, T_{max}[c] : Time to reach maximum plasma concentration, AUC[d]: Area under the plasma concentration-time curve, $t_{1/2}$[e]: Half-life, F[f]: bioavailability, CL[g]: Clearance, IV[h]: Intravenous

Supplementary Table S9: Effect of enzyme induction and inhibition on labetalol pharmacokinetics

	Vd[a] (L)	C_{max}[b](ng/ml)	AUC[c] (ng.min/ml)		$t_{1/2}$[d] (hr)		F[e] %	CL[f] (L/hr)
			Oral	IV[g]	Oral	IV		
Before Glutethimide	542 ± 72		40596 ± 11534	24438 ± 5237	3.2 ± 0.3	3.4 ± 0.5	30 ± 3	116.4 ± 30.5
After Glutethimide	509 ± 59	117 ± 46	22057 ± 6276	22447 ± 2398	2.93 ± 0.28	3.7 ± 0.23	17 ± 3	104.04 ± 13.68
Before Cimetidine:	520 ± 51	338 ± 30	51029 ± 7950	34924 ± 2288	4.3 ± 0.3	5.7 ± 0.75	25 ± 2	63.72 ± 6.96
After Cimetidine:	445 ± 24		84772 ± 19444	37943 ± 2521	4.36 ± 0.38	5.3 ± 0.5	39 ± 8	57.84 ± 6.42

Vd[a]: Volume of distribution, C_{max}[b]: Maximum plasma concentration, AUC[c]: Area under the plasma concentration-time curve, $t_{1/2}$[d]: Half-life, F[e]: bioavailability, CL[f]: Clearance, IV[g]: Intravenous

Supplementary Table S10: Average fold error (AFE) in a healthy population

$R_{obs/pre}$ of all studies	$\sum R\ ratio$	$\sum R\ ratio / N$	$Log \sum R\ ratio / N$	$10^{Log \sum R\ ratio / N}$
		**HEALTHY**		
		**IV**		
		**C_{max}[c]**		
1.56	4.35	1.45	0.161	1.44
1.19				
1.60				
		**AUC_{0-t}[d]**		
1.09	3.627	1.209	0.082	1.207
1.039				
1.498				
		**CL[e]**		
1.2	2.73	0.91	-0.040	0.912
0.9				
0.63				
		**Oral**		
		**C_{max}**		
1.13	2.39	1.195	0.077	1.193
1.26				
		**AUC_{0-t}**		
0.80	1.69	0.845	-0.073	0.84
0.89				
		**CL**		
1.18	2.11	1.055	0.023	1.054
0.93				

R[a] ratio: R_{obs}/R_{pre}, N[b]: total number of studies, C_{max}[c]: Maximum plasma concentration, AUC_{0-t}[d]: Area under the plasma concentration versus time curve, CL[e]: Clearance

Supplementary Table S11: Average fold error (AFE) in a diseased population.

$R_{obs/pre}$ of all studies	$\sum R\ ratio$	$\sum R\ ratio / N$	$Log \sum R\ ratio / N$	$10^{Log \sum R\ ratio / N}$
		**HEPATIC DISEASE**		
		**IV**		
		C_{max}[c]		
0.99	0.99	0.99	0.0043	1.009
		AUC_{0-t}[d]		
0.93	0.93	0.93	-0.03	0.933
		**CL**[e]		
1.16	1.16	1.16	0.06	1.148
		**Oral**		
		C_{max}		
2.635	2.635	2.635	0.420	2.63
		AUC_{0-t}		
1.5	1.5	1.5	0.176	1.499
		**CL**		
0.6	0.6	0.6	-0.22	0.60
		**RENAL FAILURE**		
		C_{max}		
0.9109	0.9109	0.9109	-0.040	0.9120
		AUC_{0-t}		
0.77	0.77	0.77	-0.11	0.77
		**CL**		
1.5	1.5	1.5	0.17	1.479

R[a] ratio: $R_{Obs/}R_{Pre}$, N[b]: total number of studies, C_{max}[c]: Maximum plasma concentration, AUC_{0-t}[d]: Area under the plasma concentration versus time curve, CL[e]: Clearance

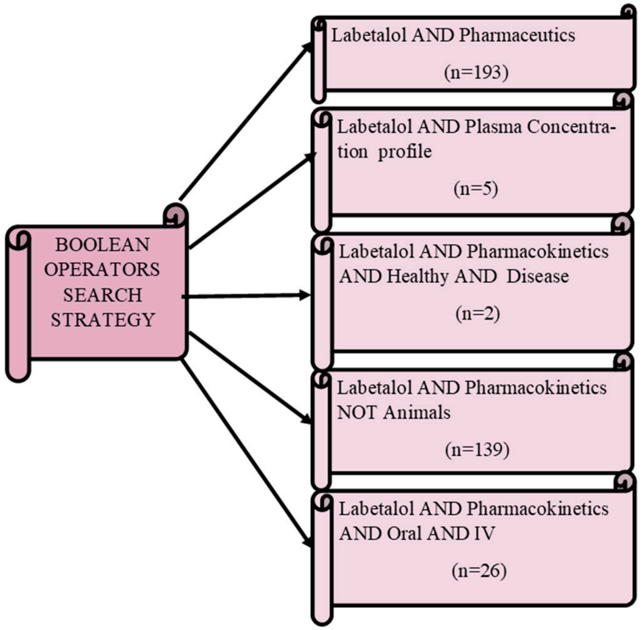

Supplementary Figure S1. Boolean Operators search strategy

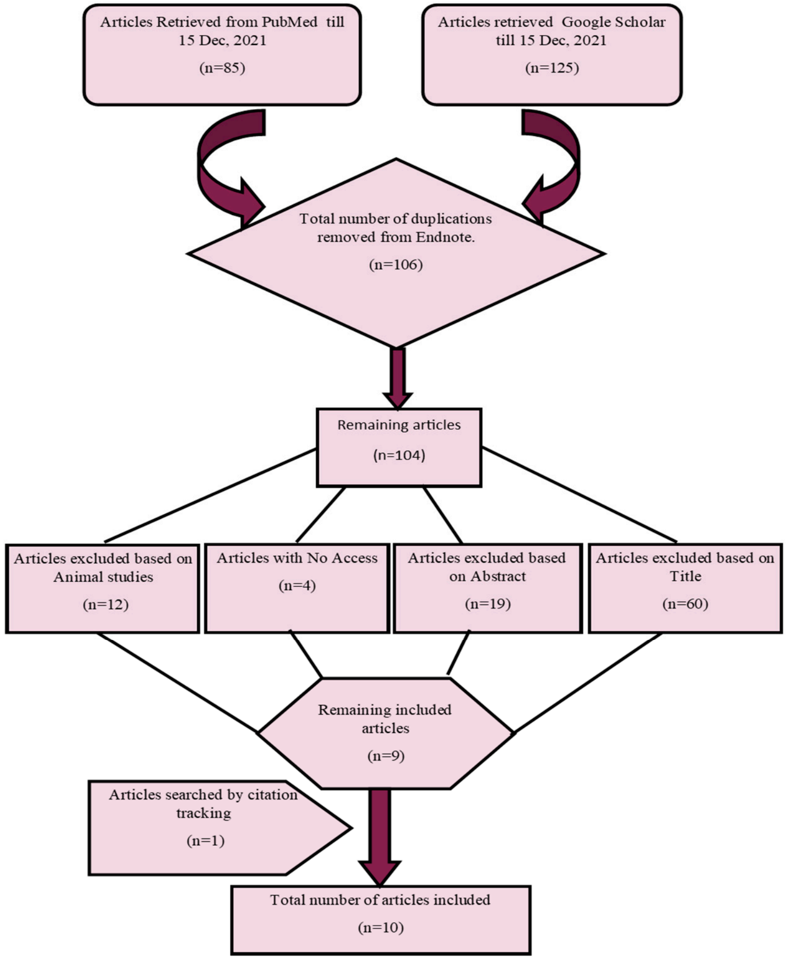

Supplementary Figure S2. PRISMA flow chat diagram
